# An extracellular mechanism that can explain the neurotoxic effects of α-synuclein aggregates in the brain

**DOI:** 10.3389/fphys.2012.00297

**Published:** 2012-07-26

**Authors:** C. Pacheco, L. G. Aguayo, C. Opazo

**Affiliations:** ^1^Laboratory of Neurobiometals, Department of Physiology, University of ConcepciónConcepción, Chile; ^2^Laboratory of Neurophysiology, Department of Physiology, University of ConcepciónConcepción, Chile

**Keywords:** Parkinson's disease, neurodegeneration, extracellular α-synuclein aggregates, plasma membrane, pore, perforation

## Abstract

Neurodegenerative diseases, such as Parkinson's disease (PD), Alzheimer's disease (AD), and Dementia with Lewy bodies (DLB), display an accumulation of proteins including α-synuclein aggregates in cortical and subcortical regions of the brain. PD is a complex, progressive disease which involves damage of motor and cognitive brain regions, as well as autonomic and sensory areas. Since α-synuclein is a neuronal cytosolic protein, it is assumed that pathogenic changes induced by α-synuclein aggregates occur only at the cytoplasmic level. However, recent studies have identified the presence of extracellular α-synuclein, suggesting that the pathogenic action of this protein may also occur in the extracellular milieu through an unknown mechanism. One of the hypotheses is that extracellular α-synuclein aggregates or oligomers may directly disrupt the neuronal membrane by the formation of a pore reminiscent to the ones formed by β-amyloid aggregates. Here, we will review some evidence that support this mechanism, analyzing the interactions of α-synuclein with components of the plasma membrane, the formation of pore/perforated structures, and the implications on ionic dyshomeostasis. Furthermore, we will also discuss how this mechanism can be integrated into a general phenomenon that may explain the synaptotoxicity and neurotoxicity observed in different neurodegenerative diseases.

## Neurodegenerative diseases and α-synuclein

Neurodegenerative diseases typically involve deposits of inclusion bodies that contain abnormal aggregated proteins (Bossy-Wetzel et al., 2004). Therefore, it has been suggested that protein aggregation is a pathogenic process, characterized by multi-steps in protein conformational changes (Ross and Poirier, 2004).

The homeostasis of proteins, or recently called *Proteostasis*, refers to different cellular pathways that regulate protein synthesis, folding, function, and degradation (Roth and Balch, 2011). There is plenty of evidence indicating that the overall proteostasis is impaired with aging, manifesting an increase in protein oxidation along with changes that exacerbate the aggregation, providing a partial explanation for several diseases known as conformational diseases (Balch et al., 2008). Neurodegenerative diseases are conformational diseases characterized by abnormal protein deposition, which can be cytoplasmic, nuclear, or extracellular (Bossy-Wetzel et al., 2004). Protein aggregates called *Amyloid* are characterized by a β-sheet secondary structure presenting higher affinity to bind dyes like Congo red and Thioflavin-T, higher resistance to proteolytic degradation, and a fibrillar appearance under electron microscopy (Ross and Poirier, 2004).

α-Synuclein is an unstructured soluble protein that can assemble into amyloid aggregates forming intracellular inclusion bodies called Lewy bodies (LBs) present in Parkinson's disease (PD), Dementia with Lewy bodies (DLB), and Multiple System Atrophy (MSA) (Spillantini et al., 1997, 1998; Tu et al., 1998). These disorders are known as synucleinopathies. However, this neuropathological feature is also commonly found in both sporadic and familial cases of Alzheimer's disease (AD). Therefore, α-synuclein dysfunction might be a common factor in several neurodegenerative diseases (Goedert, 2001).

LBs consist of eosinophilic and round cytoplasmic inclusions that are particularly enriched in the substantia nigra in PD (Braak et al., 2004), cortical brain areas in DLB (Kosaka, 1978), and amygdala in AD (Lippa et al., 1999). However, there is wide evidence that indicates that small α-synuclein species (i.e., oligomers) are responsible for the synaptotoxicity, neurotoxicity, and the final spread of this neurodegenerative disease throughout the brain (Winner et al., 2011). Therefore, the formation of α-synuclein inclusion bodies could be a protective mechanism against the smaller species. Nevertheless, it is possible that both soluble misfolded intermediates and amyloid-like fibril deposits may be toxic, but perhaps by different cellular mechanisms. For example, soluble oligomeric species in AD might affect synaptic transmission (Parodi et al., 2010), whereas LBs are intracellular space-occupying entities that may interfere with neuronal and glial intercellular communication (Goedert, 2001).

Here, we will review the progression pattern of PD and some properties of α-synuclein oligomers, especially their association to the membrane. Finally, we will propose that extracellular α-synuclein is implicated in synaptotoxicity and neurotoxicity by making the plasma membrane more permeable.

## Parkinson's disease

PD is a human motor control disorder and the second most common neurodegenerative disorder after AD (Goedert, 2001). It affects 1–2% of the adult population over 65 years and is clinically characterized by muscle rigidity, bradykinesia (slowness of movement), and tremor at rest (Jankovic, 2008). Pathologically, PD is characterized by loss of dopaminergic neurons in the *substantia nigra pars compacta* (an important region of the basal ganglia that regulates movement) (Goedert, 2001) and by the intracellular accumulation of LBs in surviving neurons and Lewy neurites that advance in a topographically predictable sequence (Braak et al., 2004).

The etiology of PD is unknown, although older age and neurotoxins are established risk factors (Marttila and Rinne, 1981; Langston et al., 1983). Actually, there is much evidence suggesting that α-synuclein aggregates participate in the pathogenesis of the disease. First, intraneuronal LBs are a histological feature of PD (Spillantini et al., 1997, 1998). Second, genetic analyses have identified three mutations of α-synuclein associated to inherited forms of PD (Polymeropoulos et al., 1997; Kruger et al., 1998; Zarranz et al., 2004), where each of the mutant variants have been shown to alter the oligomerization or fibrilization of the protein (Conway et al., 2000). Finally, it was determined that duplication or triplication of the gene locus of α-synuclein results in an increased expression of this protein, linked to a familial form of PD (Singleton et al., 2003; Chartier-Harlin et al., 2004; Ibanez et al., 2004). Overall, these findings suggest that the accumulation of α-synuclein is a critical factor in PD.

## Parkinson's progression

**Figure 1:**
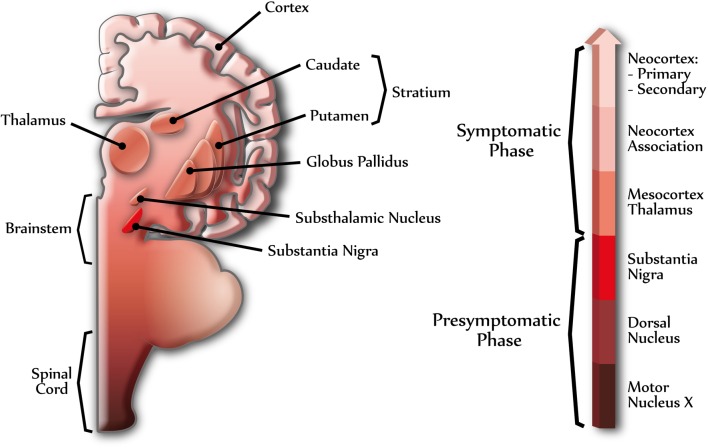
**Different stages of Parkinson's disease.** The scheme shows the progression of PD in the human central nervous system. α-Synuclein can assemble into amyloid aggregates forming Lewy bodies (LBs) and Lewy neurites, beginning at specific brain regions (dark colors) and progressing through different brain areas (light colors), in a topographically predictable sequence (Braak et al., 2004). The right arrow shows the progression of PD, indicating some of the brain regions related to presymptomatic and symptomatic phases. Dark colors indicate early stages of PD and light colors indicate late stages of PD.

PD is mainly described as a motor disease, but as the disease progresses important regions associated to autonomic, limbic, and somatomotor functions become affected (Figure 1) (Braak et al., 2004). Moreover, dementia, a non-motor symptom, is detected in a large proportion of patients with PD over the course of the disease; however, the time of onset and rate of the cognitive decline is highly variable (Aarsland et al., 2007). Dementia with LBs accounts for β30% of all age-related dementias, and up to 40% of AD cases exhibit α-synuclein pathology (Parkkinen et al., 2003; Jellinger, 2004; Mikolaenko et al., 2005; Zaccai et al., 2005). It is interesting to note that 20–40% of PD patients have cognitive impairments at disease onset and that β80% of PD patients eventually develop dementia (PDD) (Aarsland et al., 2007). In some individuals, however, cognitive decline can develop in the presence of mild PD-related cortical pathology and, conversely, widespread cortical lesions do not necessarily lead to cognitive decline (Braak et al., 2005). The mechanisms leading to the initiation and spread of α-synuclein pathology in PD are not well understood and one possibility to answer this query is that α-synuclein aggregates can progress to different brain areas in a similar manner to the propagation of prion diseases (Aguzzi, 2009). Interestingly, spread of LBs from host tissues to long-term fetal cell grafts in the brain of PD patients has been shown (Kordower et al., 2008) and supported by other studies (Mendez et al., 2005; Li et al., 2008). Therefore, it appears possible to generate the formation of LB-like structures in healthy normal neurons.

## Cell biology and biochemistry of α-synuclein

### Localization

The synuclein protein family is expressed in three forms, α-, β-, and γ-synuclein, which have multiple locations (Lavedan, 1998). Immunohistochemical studies have shown that α- and β-synuclein are mainly found in nerve terminals, in close proximity to synaptic vesicles in the central nervous system (Clayton and George, 1999). In contrast, γ-synuclein seems to be present throughout nerve cells in the peripheral nervous system (Clayton and George, 1999). Regarding cellular localization, α-synuclein is located in the cytoplasmic space, but recent studies have also identified the presence of extracellular α-synuclein (El-Agnaf et al., 2003). An analysis of α-synuclein levels in the cerebrospinal fluid (CSF) of patients clinically diagnosed with PD, progressive supranuclear palsy (PSP), AD, and age matched controls have shown that CSF α-synuclein oligomer levels are higher in the PD group as compared with the other three groups (Tokuda et al., 2010). This result indicates that α-synuclein is present in extracellular brain fluids, but most importantly, α-synuclein oligomers are enriched in the extracellular space in PD. In agreement with this, a previous report described that α-synuclein is normally released by neuronal cells and is present in human CSF and peripheral plasma (El-Agnaf et al., 2003), indicating that somehow α-synuclein may be released under physiological conditions. Moreover, these results suggest that α-synuclein might be able to act at both intra- and extracellular spaces and open the possibility that extracellular α-synuclein oligomers diffuse from one cell to another, which could explain in some way the progression of the disease to other brain regions.

### Structure

α-Synuclein has four isoforms composed by 98, 112, 126, and 140 amino acids, with the latter being the most abundant (Bisaglia et al., 2009). Under a native soluble conformation, α-synuclein is found as an unfolded protein without a stable tertiary structure belonging to intrinsically unstructured proteins. It is recognized that its conformation can change depending on several conditions (Uversky and Fink, 2002). In fact, when α-synuclein is in the presence of anionic micelles, the conformation of its N-terminal region is predominantly α-helix (Ulmer and Bax, 2005). The N-terminal domain of α-synuclein is almost entirely composed of eleven complete amino acid repeats that contain the consensus sequence, XXKTKEGVXXXX, spanning the first 89 residues of the protein, where three mutations (A30P, E46K, and A53T) associated with early development of PD are localized (Bisaglia et al., 2009). This domain is remarkably similar to a domain present in apolipoproteins, which is characterized by amphipathic α-helices (Davidson et al., 1998). With respect to the central domain of α-synuclein, it is highly amyloidogenic, rich in hydrophobic amino acids (60–95) and responsible for the aggregation and β-sheet formation (Bisaglia et al., 2009). β-Synuclein, on the other hand, lacks this domain and therefore cannot aggregate, which correlates well with studies that show that α-synuclein levels are increased in pathogenic conditions while β-synuclein levels are decreased (Rockenstein et al., 2001). This segment was identified in the brain of patients with AD and called the “non amyloidogenic component” (NAC) of senile plaques (Masliah et al., 1996). Finally, the C-terminal region present mainly acidic residues and therefore can modulate the formation of α-synuclein aggregates depending on the pH of the environment (Hoyer et al., 2002).

### Physiological role of α-synuclein

The physiological role of α-synuclein has not been determined with certainty yet, mainly because knockout mice for this protein do not present a substantial change in the phenotype, suggesting that its function is likely redundant (Chandra et al., 2004). So far, it has been determined that α-synuclein is expressed after synaptic development, suggesting that synucleins may not be critical for synapse formation (Murphy et al., 2000). Mice lacking α-synuclein were found to show increased release of dopamine at the *striatum*, indicating that this protein could function as an activity dependent negative regulator of neurotransmission in this brain region (Abeliovich et al., 2000). However, substantial evidence now indicates that α-synuclein is a key regulator for synaptic vesicle dynamics (Murphy et al., 2000; Chandra et al., 2004, 2005; Burre et al., 2010). It has been shown that α-synuclein acts as a nonclassical chaperone (Burre et al., 2010), complementing the action of CSPα (cysteine-string-protein-α), whose function is to facilitate the correct assembly of the soluble N-ethylmaleimide-sensitive factor attachment protein receptor (SNARE) complex that participates in vesicular release (Chandra et al., 2005). Another studies demonstrates that the loss of α-, β-, and γ-synuclein genes results in neuronal dysfunction as the mice age progresses (Greten-Harrison et al., 2010). Is important to know that different evidences indicates that presynaptic dysfunction act as an early event of neurodegeneration (Gray et al., 2009; Nemani et al., 2010), therefore a failure of the maintenance of SNARE protein function in neurotransmitter release could lead to PD (Sharma et al., 2012), which could be due to a dysfunction of α-synuclein (Burre et al., 2010; Greten-Harrison et al., 2010; Nemani et al., 2010).

### Pathological role

Since α-synuclein is a cytosolic protein, it is assumed that the principal pathological alterations induced by this protein occur only at the intracellular level. Among the mechanisms that explain the role of intracellular α-synuclein on PD pathogenesis, the following are well known: (1) inhibition of the ubiquitin-proteasome system (Emmanouilidou et al., 2010), (2) changes in synaptic vesicle release (Murphy et al., 2000), (3) mitochondrial dysfunction (Elkon et al., 2002), (4) pore formation (Volles et al., 2001; Quist et al., 2005), and (5) production of reactive oxygen species (Junn and Mouradian, 2002). All these alterations would eventually lead to neuronal dysfunction and death (Cookson and van der Brug, 2008). However, recent studies have identified the presence of extracellular α-synuclein (El-Agnaf et al., 2003, 2006), suggesting that the pathogenic action of this protein may also take place in the extracellular space (Lee et al., 2008; Lee, 2008; Luk et al., 2009).

It is possible that α-synuclein has a dual role: a physiological role involved in the regulation of synaptic transmission; and a pathological role associated with neurodegenerative processes. Can these two roles be sequentially/temporally/directly connected? There is still no clear answer to these questions, but we think that there must be a synaptic switch that commands the onset of the disease. So it is important to understand what are the differences and similarities between the pathogenic and physiological mechanisms of α-synuclein. Part of the answer might lay in the structural flexibility that α-synuclein displays which facilitates the transition from a soluble (physiological) to an aggregate state (pathogenic).

### α-synuclein aggregates

With respect to the process of α-synuclein aggregation, *in vitro* studies indicate the presence of a nucleation-dependent mechanism characterized by a slow onset (Cremades et al., 2012) and a subsequent growth phase culminating in a steady state (Wood et al., 1999). This depends on the nature of α-synuclein (wild-type or mutant) (Li et al., 2001), as well as the incubation conditions such as pH, temperature (Uversky et al., 2001a), concentration of metal ions (Uversky et al., 2001b), and other agents such as pesticides (Uversky et al., 2001c). Structurally, the aggregates *in vitro* closely resemble aggregates from brain exhibiting typical amyloid fibril morphology (Conway et al., 2000). New evidence suggests that wild-type α-synuclein forms a stably folded tetramer that resists aggregation (Bartels et al., 2011). On the basis of these recent findings, it has been proposed that destabilization of the helically folded tetramer precedes α-synuclein misfolding and aggregation in PD. Mutations associated to PD, both A53T and A30P, promote the formation of prefibrilar oligomeric species, while E46K reduces the formation of these aggregates (Bisaglia et al., 2009). Additionally, potentially toxic species appear to correspond to low molecular weight species such as dimers, trimers, tetramers, pentamers, and hexamers, which have molecular weights between 20–100 kDa (Danzer et al., 2007). The mechanism through which α-synuclein monomers are converted into toxic oligomers and then fibers is largely unknown (Tsigelny et al., 2007), but it is believed that this process initiates at the plasma membrane in lipid raft micro domains (Bar-On et al., 2008).

### α-synuclein secretion

**Figure 2:**
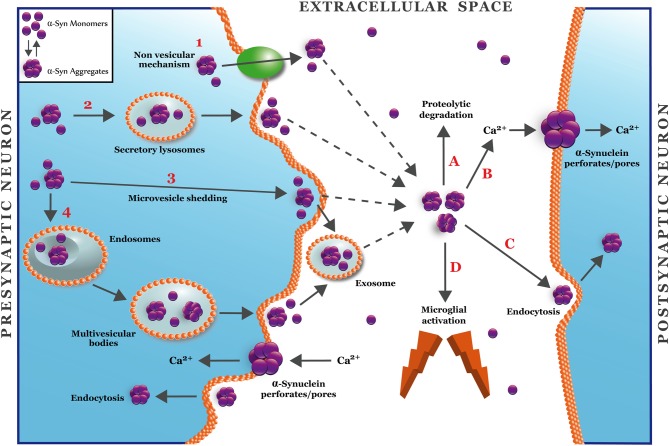
**A hypothetical model for secretion and synaptic targets of α-synuclein oligomers.** The figure shows four different mechanisms for unconventional secretion of α-synuclein (Nickel and Rabouille, 2009). Mechanism 1 depicts a non-vesicular translocation of α-synuclein mediated by an unknown transmembrane protein located at the plasma membrane. Mechanisms 2–4 depict vesicular mechanisms for α-synuclein secretion that involve lysosomes (2), microvesicle shedding (3), or endosome/multivesicular bodies (4). Mechanisms 3 and 4 involve the release of α-synuclein into exosomes. Once in the extracellular space (Lee, 2008), α-synuclein oligomers can be removed by proteolytic degradation (A) or endocytocis (C) (Desplats et al., 2009). The accumulation of α-synuclein in the extracellular space can activate microglia (D) or alter the plasma membrane through the formation of pore/perforations (B) that can deregulate calcium and metal transition homeostasis (Danzer et al., 2007).

How is α-synuclein secreted? Although the exact mechanism has not been characterized yet, it has been found that a small portion of α-synuclein is secreted into the extracellular medium via unconventional exocytosis, different to the classical exocytosis through ER/Golgi (Lee et al., 2005), which is expected since α-synuclein lacks an ER targeting signal peptide (Lee et al., 2005). The mechanism of the nonconventional exocytosis is not well understood; therefore it could be more than one mechanism to explain this new pathway (Nickel and Rabouille, 2009). The model presented in Figure 2 describes several ways to explain the secretion of soluble and aggregated forms of α-synuclein. The α-synuclein secretion would occur under physiological conditions although it would be more prone under pathological conditions, such as those associated to mitochondrial and proteosomal dysfunction, which are associated with PD (Lee et al., 2005). It was reported that α-synuclein has a higher aggregation rate into secretory vesicles compared to cytosolic α-synuclein (Lee et al., 2005). Two main hypotheses are considered for this phenomenon: (1) α-synuclein is incorporated within the vesicles by unknown mechanisms. Once the protein is in the vesicles, it increases the aggregation due to the acidic pH (Hoyer et al., 2004); or (2) pre-aggregated α-synuclein is translocated to secretory vesicles forming aggregates that are subsequently released into the extracellular medium, where it could activate a pathological process. In fact, extracellular α-synuclein aggregates induce microglial activation, cytokine release, cell death, and plasma membrane permeabilization (Feng et al., 2010; Van Rooijen et al., 2010). Moreover, this extracellular α-synuclein could be taken up through endocytosis to favor the formation of LB-like inclusions in neighboring cells, which could explain the neuropathological progression of PD (Desplats et al., 2009).

## Role of Ca^2+^ in parkinson's disease

Ca^2+^ has critical roles in neuronal functions, including the regulation of neurite outgrowth and synaptogenesis, synaptic transmission and plasticity, and cell survival (Mattson, 2007). There is a lot of evidence indicating the involvement of Ca^2+^ in PD. Some studies indicate that α-synuclein controls the function of voltage gated calcium channels (Adamczyk and Strosznajder, 2006; Hettiarachchi et al., 2009), while other studies indicate that this protein is able to increase the levels of intracellular Ca^2+^, probably due to alterations in the membrane, leading to neuronal death (Danzer et al., 2007). This is consistent with studies in human neural cells overexpressing mutant α-synuclein, which have higher plasma membrane ion permeability and basal level of intracellular Ca^2+^ compared to control cells (Furukawa et al., 2006). Interestingly, it was recently found that Aβ aggregates (implicated in AD) were able to form structures that act as pore/perforations in the neuronal membranes (Sepulveda et al., 2010). This action induced an acute increase in intracellular Ca^2+^ and a decrease in synaptic proteins in neurons chronically treated with Aβ aggregates (Parodi et al., 2010; Sepulveda et al., 2010). Based on the fact that other amyloidogenic proteins like Aβ can be inserted into the membrane and form perforations (Sepulveda et al., 2010), this might be one of the mechanism by which extracellular α-synuclein causes neurodegeneration, i.e., pore/perforation formation in the plasma membrane leading to an increase in free intracellular Ca^2+^ concentration with subsequent cell death. This idea is supported by another study showing that cells expressing α-synuclein and treated with Aβ displayed increased current amplitudes and calcium influx consistent with the formation of cation channels (Tsigelny et al., 2008a).

## Formation of α-synuclein pores/perforations

One of the first steps in the formation of pores/perforations is the association of α-synuclein to the membrane, which is discussed in the following two sections:

### α-synuclein association to membranes

It has been shown that α-synuclein associates with phospholipid bilayers in artificial membranes, especially with those membranes containing acidic phospholipids (Davidson et al., 1998), since the lipophilic N-terminal region of α-synuclein is positively charged at physiological pH (Rhoades et al., 2006). Furthermore, this association could be stabilized by increasing the α-helical secondary structure of α-synuclein (Davidson et al., 1998; Jo et al., 2000; Eliezer et al., 2001; Chandra et al., 2003). Another critical factor for the association of α-synuclein to membranes is the membrane size. It has been shown that α-synuclein preferentially associates to vesicles with small diameters (20–25 nm) (Davidson et al., 1998). On the other hand, recent studies demonstrate that the interaction of α-synuclein with vesicular membranes is fast and reversible and the binding of α-synuclein to neutral and negatively charged membranes occurs by apparently different mechanisms (Shvadchak et al., 2011).

In natural membranes, α-synuclein has been shown to associate, as a peripheral membrane protein, with synaptic vesicles (Maroteaux et al., 1988), axonal transport vesicles (Jensen et al., 1998), lipid droplets (Cole et al., 2002), and yeast vesicles (Outeiro and Lindquist, 2003). It has been demonstrated that α-synuclein binding occurs at the outer membrane leaflet of the plasma membrane especially in lipid rafts, domains rich in cholesterol, and sphingolipids (Fortin et al., 2004; Bar-On et al., 2008). These micro domains are characterized by slow lateral diffusion of the lipid acyl chains as well as detergent resistance (Allen et al., 2007). Moreover, lipid rafts are involved in the interaction between lipids and hydrophobic residues of the amphipathic α-helix present in the N-terminal of α-synuclein (Tsigelny et al., 2007). Deletion of the N-terminal residues leads to attenuations of α-synuclein toxicity toward yeast, suggesting that the toxicity depends on the N-terminal membrane-binding domain (Vamvaca et al., 2009). This was confirmed by point mutations in the N-terminal site of α-synuclein (A30P, E46K, and A53T) associated with familial PD (Polymeropoulos et al., 1997; Kruger et al., 1998; Zarranz et al., 2004).

### Models of α-synuclein pores/perforations

**Figure 3:**
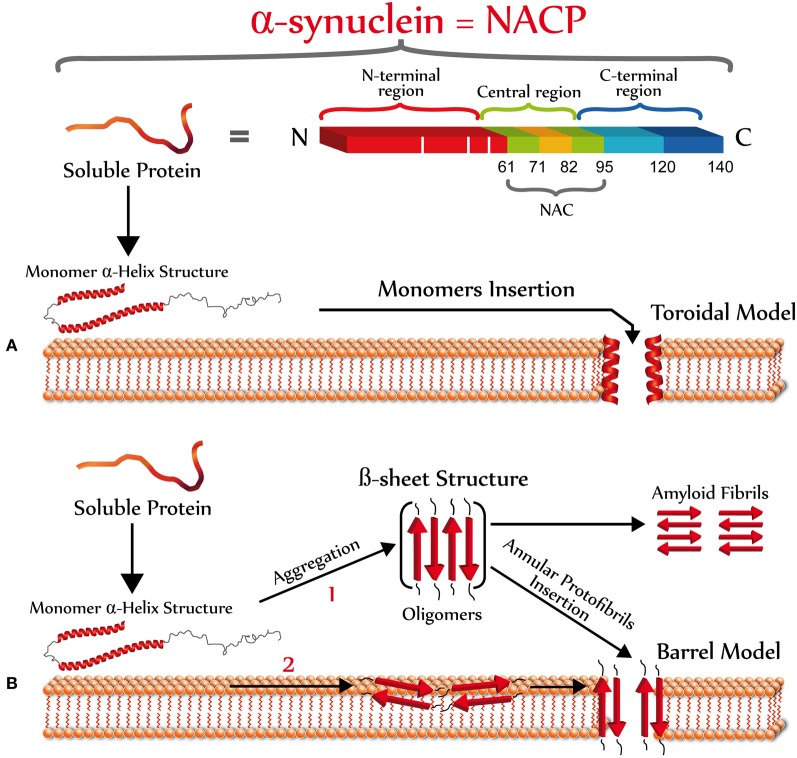
**Primary and secondary structure of α-synuclein.** Top scheme shows the three main domains of α-synuclein. The N-terminal (red) is an amphipathic domain that contains the three point mutations (white bars) linked to the autosomal dominant form of PD (Bisaglia et al., 2009). The central region (green-pale brown) is a highly hydrophobic domain that was originally identified in patients with AD or LBD, which is the precursor of the non amyloidogenic component of the extracellular senile plaque (NAC), which promotes the protein aggregation (Ueda et al., 1993). The C-terminal domain (blue) has an acidic character, which possesses anti-amyloidogenic properties (Hoyer et al., 2004). According to the operational model of membrane perforation, once α-synuclein interacts with the plasma membrane, it should acquire a new secondary structure to form pores/perforations. The formation of α-synuclein pores may be explained by two different models: **(A)** A toroidal model involving a sequential binding of α-synuclein monomers to the plasma membrane that results in the formation of pores or channels with α-helical conformation (Zakharov et al., 2007); and **(B)** A barrel model showing that α-synuclein oligomers (β-sheet-rich) form ring structures with a central pore (Volles and Lansbury, 2003). The formation of α-synuclein oligomers enriched in β-sheet structures could occur in the extracellular space (1) or at the plasma membrane (2).

One of the biggest complications in understanding the structure of the α-synuclein pore is that there are no crystallographic data for this protein, likely due to the complexity to crystallize amyloid species. There is also a lack of high-resolution models for biological membranes that would aid in the understanding of the mechanism by which α-synuclein pores/perforations are formed. However, there are mainly two types of pore models, toroidal and barrel (Yang et al., 2001), that may explain α-synuclein perforation properties (Figure 3). A toroidal model involves a sequential binding of α-synuclein monomers to lipid membranes that result in the formation of pores or channels with α-helical conformation (Zakharov et al., 2007). In fact, it is believed that the basic KXKE repetitions in the N-terminal of α-synuclein act as a membrane voltage sensor (Zakharov et al., 2007). Furthermore, *in silico* studies have shown that the sequential binding of α-synuclein to the membrane occurs between the N-terminal of each of the monomers, leading to the formation of pentamers and hexamers to generate ring-type structures with different external size ranges (9–15 nm) and internal diameters (2–5 nm) (Tsigelny et al., 2007). Even aged samples of α-synuclein, with an expected β-sheet oligomeric conformation, were shown to bind vesicles in an α-helical state (Smith et al., 2008). This model contrasts with the barrel model that indicates that α-synuclein oligomers (β-sheet-rich) form ring structures with a central pore (Volles and Lansbury, 2003). These aggregates would bind in the membrane (Volles et al., 2001) causing its permeabilization in a concentration-dependent manner, which has been shown in liposomes (Volles et al., 2001) and artificial membrane bilayers (Kayed et al., 2004). Moreover, using atomic force microscopy (AFM), it was found that α-synuclein in artificial membranes form pores with an outer diameter of 8–10 nm and an inner diameter of 1–2 nm (Quist et al., 2005). Interestingly, this type of structure is very similar to the structures formed by pore forming proteins such as amylin (Quist et al., 2005) and gramicidin (Diociaiuti et al., 2002). Analysis of single channel currents in artificial membranes indicated that α-synuclein aggregates form pores with different conductances, suggesting that different α-synuclein oligomeric species are involved in the formation of these membrane structures (Quist et al., 2005). In addition, studies performed with the α-synuclein mutants, A53T, and A30P; suggest that PD-associated mutations may affect the architecture of the α-synuclein pore/perforations (Furukawa et al., 2006). This pore-like activity may only be associated with α-synuclein protofibrils, since β-synuclein protofibrils (also enriched in β-sheet structures) are apparently not able to permeate or bind to lipid vesicles (Park and Lansbury, 2003). These permeabilizing pore-like structures should only allow the selective transport of molecules according to the molecular diameter. For example, both Ca^2+^ and dopamine pass through the membrane in the presence of α-synuclein protofibrils, while larger molecules such as cytochrome c do not (Volles et al., 2001). On the other hand, it has been shown that these pores or perforated structures are sensitive to Zn^2+^ (Tsigelny et al., 2007), likely as a result of the interaction between Zn^2+^ and cysteine, histidine or arginine residues of α-synuclein. Specifically, the histidine residue 50 of the α-synuclein monomer is considered as a possible candidate for interaction with Zn^2+^, mainly because it is located near the site of the putative pore (Zakharov et al., 2007). The above is consistent with studies in human cells overexpressing mutant α-synuclein whose ionic permeability is affected by chelation of divalent cations (Furukawa et al., 2006).

## Conclusions

In conclusion, we reviewed information that supports the role of extracellular α-synuclein in PD. Like other prone-aggregating proteins, α-synuclein can interact with lipids in the plasma membrane, increasing membrane permeability by the formation of not well characterized structures (pore or perforations) that may lead to calcium or transition metal dyshomeostasis, which may induce subsequent synaptotoxicity and neuronal death.

α-Synuclein protein is the main component of LBs present in PD, DLB, and related neurodegenerative diseases. Therefore, α-synuclein aggregates (intracellular and extracellular) could be enhancing the synaptotoxic/neurotoxic effects of other protein aggregates (oligomers) such as Aβ aggregates. In fact, recent studies have shown that Aβ and α-synuclein form hybrid pore-like oligomers that could change membrane permeability (Tsigelny et al., 2008b).

However, further studies are needed to understand the precise mechanisms by which extracellular α-synuclein forms channels, pores, or perforations and the role that these structures have in neurodegenerative diseases, especially in PD. A crucial question is what α-synuclein species are able to disrupt the plasma membrane? Future experiments should identify these structures and determine if they are potential targets for pharmacological therapies.

Currently, we believe that studies focused on unraveling the mechanism of the formation of α-synuclein pore/perforations will provide new insights for the generation of innovative approaches to interfere with these synaptotoxic/neurotoxic processes initiated at the neuronal membrane in order to develop new therapeutic strategies for treating people with PD and other neurodegenerative diseases.

### Conflict of interest statement

The authors declare that the research was conducted in the absence of any commercial or financial relationships that could be construed as a potential conflict of interest.

## Abbreviations

AD: Alzheimer's disease
LBs: Lewy bodies
PD: Parkinson's disease.
